# T42. WHEN SHOULD EARLY INTERVENTION START, AND FOR HOW LONG SHOULD IT LAST?

**DOI:** 10.1093/schbul/sby016.318

**Published:** 2018-04-01

**Authors:** Nikolai Albert, Marianne Melau, Heidi Jensen, Lene Halling Hastrup, Carsten Hjorthøj, Merete Nordentoft

**Affiliations:** 1 Mental Health Center Copenhagen; 2 Region Hovedstadens Psykiatri; 3 Psychiatric Research Unit, Region Zeal

## Abstract

**Background:**

Early intervention in psychosis facilities have often failed to integrate the two main elements of early intervention. While some facilities have focused on early, and have had Duration of Untreated Psychosis (DUP) as their main target, others have focused on the intervention and the treatment provided when patients were diagnosed. As both DUP reduction and specialized early intervention (SEI) has proved to have an effect on the treatment of patients with first-episode psychosis one could hope of a synergetic effect if the two strategies were integrated. In this study, we use data from a randomized clinical trial testing the effect of prolonged early intervention (5 years) compared to standard specialized early intervention (2 years). Overall the study found that both treatment groups remained stable or improved in psychopathology, functioning and cognition and that there was no further beneficial effect of the prolonged the treatment. Participants had a long DUP (median 52 weeks). For this specific sub-study we hypothesized that patients who were treated early in their course of illness would have a beneficial effect of the prolonged treatment compared to those who only received 2 years of specialized treatment.

**Methods:**

296 participants with a psychotic diagnosis within the schizophrenia spectrum (ICD 10 – F2x, excluding F21) were included. DUP start was assessed from first psychotic symptom equivalent to 3 or above on a global SAPS item. DUP stop was when patients started antipsychotic treatment or specialized early intervention treatment. To assess if there were a delay within the mental health referral system we used the national register to identify when participants first were diagnosed with a schizophrenia spectrum diagnosis and calculated the time until they started SEI treatment. Finally, we added the DUP and the treatment delay together to assess the time from first psychotic symptom until the start of adequate treatment (both antipsychotic medication and specialized early intervention treatment), called total treatment delay. We analyzed if there were a treatment effect for participants with DUP shorter than 3 months (n=79) and if there were an effect for participants with a total treatment delay shorter than 6 months (n=54). We used multiple imputations to correct for missing data at the follow-up. The data were analyzed using binary logistic regression for the dichotomous variables and linear regression for the continuous variables.

**Results:**

At the five year follow-up, the participants who had a short DUP and had received 5 years of SEI treatment had lower psychopathological scores and higher level of functioning and cognition than those who only received 2 years of SEI treatment. The difference was not significant. For the patients who had a short total treatment delay there was a clear trend favoring the prolonged treatment and for negative symptoms there was a near significant effect of the prolonged treatment (estimated mean difference -0.61, 95% CI -1.2;0.006, p=0.05).

**Discussion:**

Our findings are results of a sub-group analysis and should be interpreted with caution. Even if the results from the main trail did not find a significant effect of prolonged SEI treatment this sub-group analysis indicates that some of the explanation could be the delay prior to the start of treatment and that there could be a beneficial effect of the prolonged treatment if it actually were provided within the early years of illness and not just in the early years after diagnosis.

